# Supplementary material for: Human-Derived Cortical Neurospheroids Coupled to Passive, High-Density and 3D MEAs: A Valid Platform for Functional Tests
Source: Bioengineering (Basel). 2023 Apr 6;10(4):449. doi: 10.3390/bioengineering10040449 (PMC10136157; doi:10.3390/bioengineering10040449)
Supplement: Supplementary file 1 [file bioengineering-10-00449-s001.zip › bioengineering-2290300-supplementary.pdf]

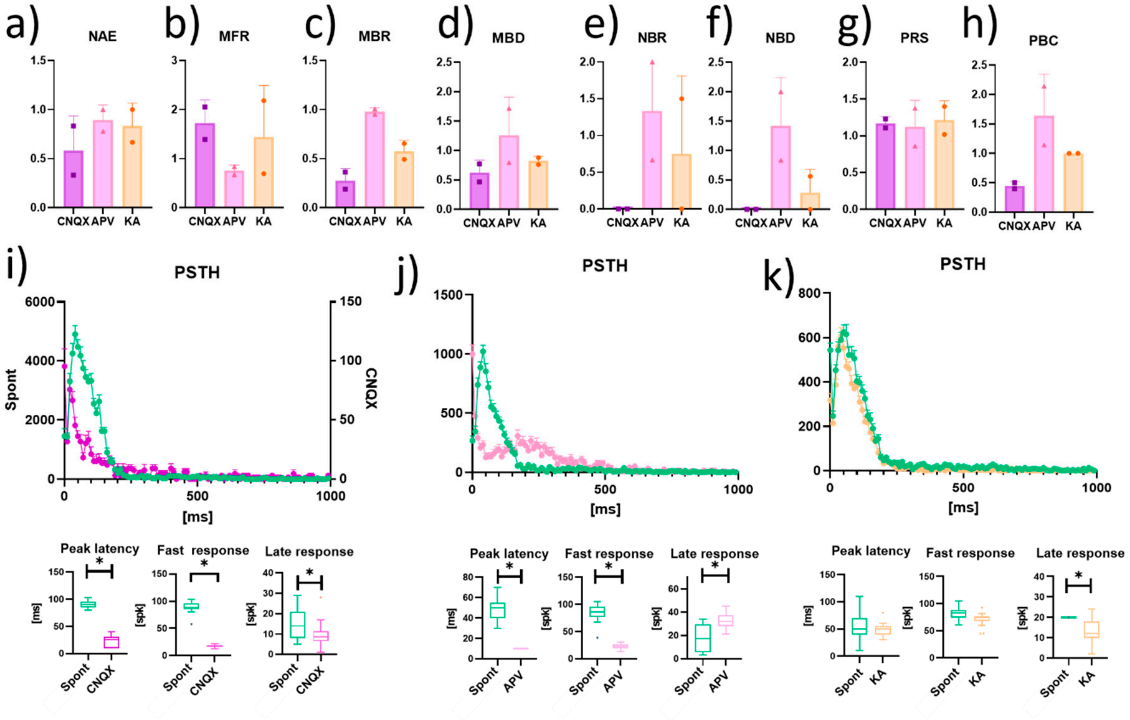

Suppl. Figure S1. Evaluation of the electrical and chemical modulation (50 µM CNQX, 80 µM APV, and 5 µM KA). Data are obtained from experiments on the MEA60 (n = 2) and are normalized to the value detected in spontaneous conditions in the absence of drugs. **(a–h)** Graphs showing the **(a)** Number of active electrodes (NAE), **(b)** mean firing rate (MFR), **(c)** Mean bursting rate (MBR), **(d)** mean burst duration (MBD), **(e)** network burst rate (NBR), **(f)** network burst duration (NBD), **(g)** percentual random spikes (PRS), and **(h)** percentual bursting channels (PBC) in neurospheroids treated with CNQX, APC, and KA, represented in purple, pink, and orange, respectively. Data are represented as a mean and standard deviation of the mean. **(i–k)** Graphs showing the effect of the modulation on the response to the electrical stimulus induced by **(i)** CNQX, **(j)** APV, and **(k)** KA. In each panel, the Post-Stimulus Time Histogram (PSTH) showing the response of neurospheroids to the stimulation is shown (not treated, treated with CNQX, APV, and KA are represented in green, purple, pink, and orange, respectively). The peak latency represents the mean latency to reach the maximum peak in the PSTHs, the fast response box plot represents the number of evocated spikes in the first 100 ms of the PSTH, while the late response box represents the number of evocated spikes between 100 ms and 1000 ms. Asterisks indicate $p<0.05$.
